# Nitro-Oleic Acid in Seeds and Differently Developed Seedlings of *Brassica napus* L.

**DOI:** 10.3390/plants9030406

**Published:** 2020-03-24

**Authors:** Martin Vollár, Gábor Feigl, Dóra Oláh, Attila Horváth, Árpád Molnár, Norbert Kúsz, Attila Ördög, Dezső Csupor, Zsuzsanna Kolbert

**Affiliations:** 1 Institute of Pharmacognosy, Faculty of Pharmacy, University of Szeged, H-6701 Szeged, Hungary; vollar@pharmacognosy.hu (M.V.); horvath.attila@pharmacognosy.hu (A.H.); k.norbi@pharmacognosy.hu (N.K.); csupor.dezso@pharmacognosy.hu (D.C.); 2 Department of Plant Biology, Faculty of Science and Informatics, University of Szeged, H-6726 Szeged, Hungary; feigl@bio.u-szeged.hu (G.F.); olah.dora18@citromail.hu (D.O.); aordog@bio.u-szeged.hu (A.Ö.)

**Keywords:** *Brassica napus*, germination, nitro-oleic acid, nitric oxide, seedlings, seeds

## Abstract

Similar to animals, it has recently been proven that nitro-fatty acids such as nitro-linolenic acid and nitro-oleic acid (NO_2_-OA) have relevant physiological roles as signalling molecules also in plants. Although NO_2_-OA is of great therapeutic importance, its presence in plants as a free fatty acid has not been observed so far. Since *Brassica napus* (oilseed rape) is a crop with high oleic acid content, the abundance of NO_2_-OA in its tissues can be assumed. Therefore, we quantified NO_2_-OA in *B. napus* seeds and differently developed seedlings. In all samples, NO_2_-OA was detectable at nanomolar concentrations. The seeds showed the highest NO_2_-OA content, which decreased during germination. In contrast, nitric oxide (•NO) levels increased in the early stages of germination and seedling growth. Exogenous NO_2_-OA treatment (100 µM, 24 h) of *Brassica* seeds resulted in significantly increased •NO level and induced germination capacity compared to untreated seeds. The results of in vitro approaches (4-Amino-5-methylamino-2′,7′-difluorofluorescein (DAF-FM) fluorescence, •NO-sensitive electrode) supported the •NO liberating capacity of NO_2_-OA. We observed for the first time that *Brassica* seeds and seedlings contain free NO_2_-OA which may be involved in germination as an •NO donor as suggested both by the results of exogenous NO_2_-OA treatment of seeds and in vitro approaches. Due to their high NO_2_-OA content, *Brassica* sprouts can be considered as a good source of dietary NO_2_-OA intake.

## 1. Introduction

Nitro-fatty acids (NO_2_-FA) as endogenous signal molecules in animals and humans have gained great attention, since these nitrated lipid derivatives exert relevant bioactivity in association with anti-thrombotic, cytoprotective, and anti-inflammatory processes (recently reviewed in Ref. [1]). The addition reaction of nitric oxide (•NO) and •NO-derived higher oxides of nitrogen (peroxynitrite, nitrogen dioxide) with conjugated double bond-containing, unsaturated fatty acids results in the formation of NO_2_-FA; although, the in vivo mechanism is still unknown [2]. During the first proposed mechanism, radical hydrogen abstraction from a bis-allylic carbon takes place resulting in the formation of an alkyl radical which is followed by the formation of a peroxyl radical via double bond rearrangement and molecular oxygen insertion. The insertion of nitrogen dioxide (•NO_2_) and the consequent formation of a non-electrophilic nitroalkane-alkene product is also possible [3]. The second mechanism includes the formation of a carbon-centred radical as the result of the direct addition of •NO_2_. This can be followed by further oxidation steps yielding the electrophilic nitro-alkene [4].

Although the occurrence and bioactivity of nitrated fatty acids is well-characterized in animals, these interesting molecules have recently been the focus of attention in plants. The presence of endogenous NO_2_-FAs such as nitro-conjugated linoleic acid (NO_2_-cLA) and nitro-oleic acid-cysteine (NO_2_-OA-Cys) adducts have been detected in extra-virgin olive oil (EVOO) and fresh olives [5]. By incubating EVOO with nitrating agents, Fazzari et al. [4] was able to detect nitro-linolenic acid (NO_2_-Ln) and nitro-linoleic acid (NO_2_-LA). Later, in *Arabidopsis thaliana* at different developmental stages [6], in pea roots and leaves as well as in rice leaves, NO_2_-Ln proved to be the major endogenous NO_2_-FA [7]. Recently, Di Palma et al. [8,9] quantified NO_2_-OA in tomato cell suspensions treated with exogenous NO_2_-OA supporting the internalization of this nitrated derivative in tomato cells.

Nitro-fatty acids show electrophilic reactivity towards cellular nucleophilic targets such as reduced glutathione or protein cysteine (Cys) and histidine (His) residues. This results in the reversible formation of protein-NO_2_-FA adducts, which serve as a reservoir and also control the size of the free NO_2_-FA pool. The fast adduction of NO_2_-OA to thiol containing proteins and to glutathione has been revealed in mice plasma [10], while recently NO_2_-OA-glutathione adducts have been identified in tomato cell suspension [8].

At the same time, the electrophilic reactivity causes post-translational modifications of Cys and His containing proteins affecting their distribution and/or function [11]. These nitroalkylation reactions are partly responsible for the biological role of NO_2_-FA [12]. Additionally, NO_2_-FA have been reported to release •NO in aqueous environments thus acting as endogenous •NO donors and transducing the •NO signal [13,14,15,16]. The signalling role of NO_2_-FA (especially NO_2_-Ln) was further supported by the fact that NO_2_-Ln content was modified during plant development and by abiotic stress [6]. Moreover, transcriptomic analysis revealed several genes, the expression of which was positively or negatively modulated by NO_2_-Ln, indicating the signalling role of NO_2_-Ln in plant cells [6]. Recently, the signalling role of NO_2_-OA has also been revealed since it induces the production of reactive oxygen species possibly through the reduction of glutathione pool or via the activation of NADPH oxidase and triggers plant defence responses in tomato cells [8,9].

Due to its role in animal signal transduction [17,18,19] NO_2_-OA is the most intensively studied NO_2_-FA with potent anti-inflammatory effects [20]. Studies in animal models pointed out the protective role of NO_2_-OA in cardiovascular, renal, and metabolic diseases [10,21,22], therefore its therapeutic application is promising. Identifying plant-derived dietary sources of NO_2_-FAs, specially the well-studied NO_2_-OA and its beneficial effects, would constitute a suitable way to access to the valuable properties described for these relevant molecules.

*Brassica* sprouts are rich sources of nutrients, phytochemicals, vitamins, minerals, enzymes, amino acids, and fatty acids, therefore they have high nutritional value [23]. The most abundant fatty acids in *Brassica* sprouts are oleic, linoleic, linolenic, erucic, palmitic, and stearic acids which account for 89–94% of the total fatty acid content [24]. *Brassica napus* (oilseed rape) seedlings are especially rich in oleic acid (61% of the total fatty acids, [24]), but the endogenous presence of NO_2_-OA in this species as well as in other plants has not been observed so far. Therefore, our aim was to examine the presence of NO_2_-OA in differentially developed *Brassica* plants. Furthermore, we aimed to examine the putative biological functions of NO_2_-OA in *Brassica* seeds and seedlings.

## 2. Results and Discussion

### 2.1. Characterization of the Synthesized NO_2_-OA Standard

**Figure 1:**
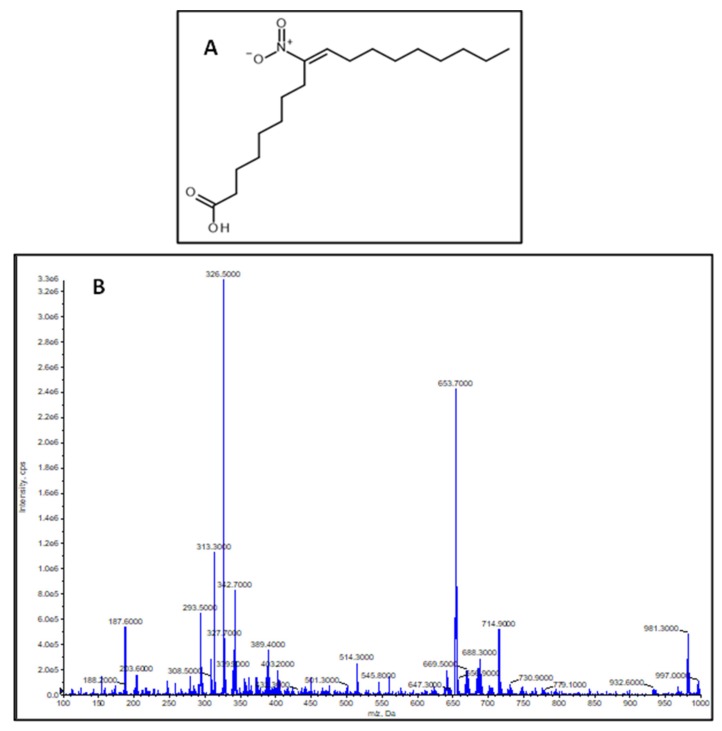
(**A**) Structure of (*E*)-9-nitrooctadec-9-enoic acid. (**B**) Q1 MS scan of nitro-oleic acid (NO_2_-OA) standard. Scan range was set between *m*/*z* 100 and 1000 Da. Molecule ion peak [M − H]^−^ was *m*/*z* 326.5.

The synthesized compound was identified as (*E*)-9-nitrooctadec-9-enoic acid based on the identical ^1^H and ^13^C NMR data with those reported in the literature [25]. This was assured by mass spectrometric measurements where the measured molecule ion mass was shown to be *m*/*z* 326.5, calculated neutral molecule mass was 327.4589 Da, molecular formula C_18_H_33_NO_4_ (Figure 1A,B).

### 2.2. Calibration

**Figure 2:**
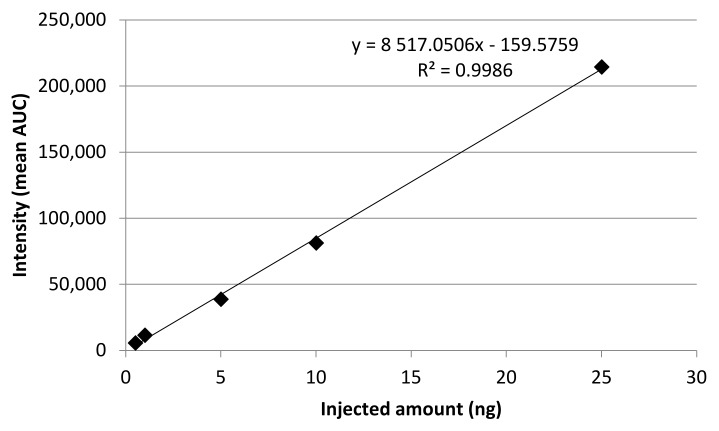
Calibration line of NO_2_-OA standard. Axis X shows the range of injected NO_2_-OA amount (ng) from working solutions, axis Y shows the mean area under curve (AUC) of the working solutions.

From the synthesized standard a stock standard was prepared with methanol (HPLC grade) and working solutions were prepared (50–2500 ng/mL) by diluting the stock standard. Each solution was injected three times, the precision (Relative Standard Deviation, RSD, %) of the calibration measurements ranged between 1.2093–2.3633%. Calibration showed linear regression, the R^2^ value was 0.9986, the limit of detection (LOD, S/N = 3.3) was 0.1184 nmol/mL, and the limit of quantitation (LOD, S/N = 10) was 0.3588 nmol/mL (Figure 2).

### 2.3. NO_2_-OA Content of Brassica napus at the Seed and Seedling Stages

**Figure 3:**
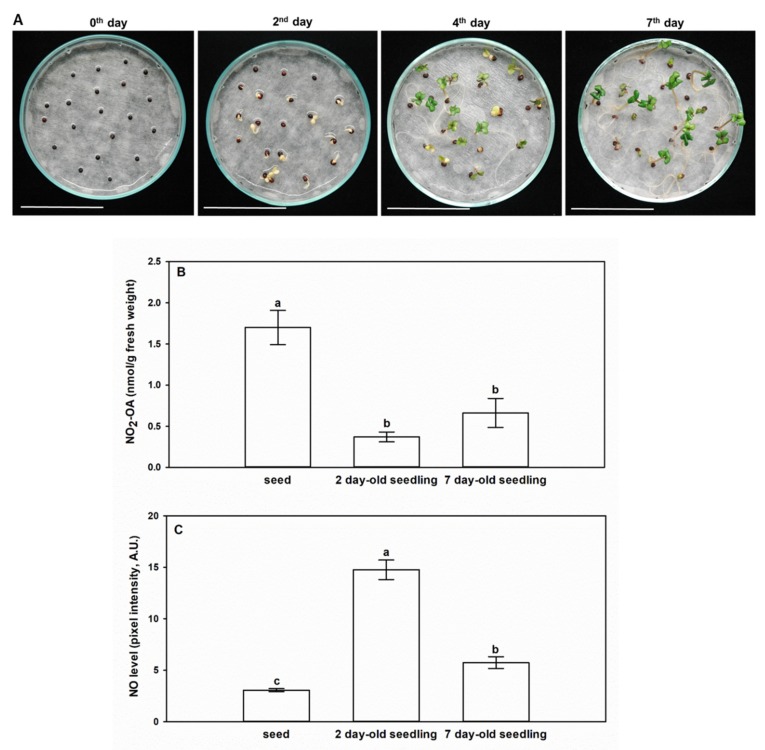
Levels of NO_2_-OA and •NO change in opposite ways in the early stage of germination (day 0) and during seedling growth (2nd, 4th and 7th days). (**A**) Representative photographs taken from *Brassica napus* seedlings on day 0, and 2nd, 4th, and 7th day after sowing. Bars = 4.5 cm. (**B**) Mean concentrations of NO_2_-OA (nmol/g fresh weight, with standard errors) in seeds and differently developed seedlings of *Brassica napus*. (**C**) •NO levels (pixel intensity, arbitrary unit) in *Brassica napus* seeds, 2 day-old and 7 day-old seedlings. Different letters indicate significant differences according to the Holm–Sidak test (P ≤ 0.001).

**Figure 4:**
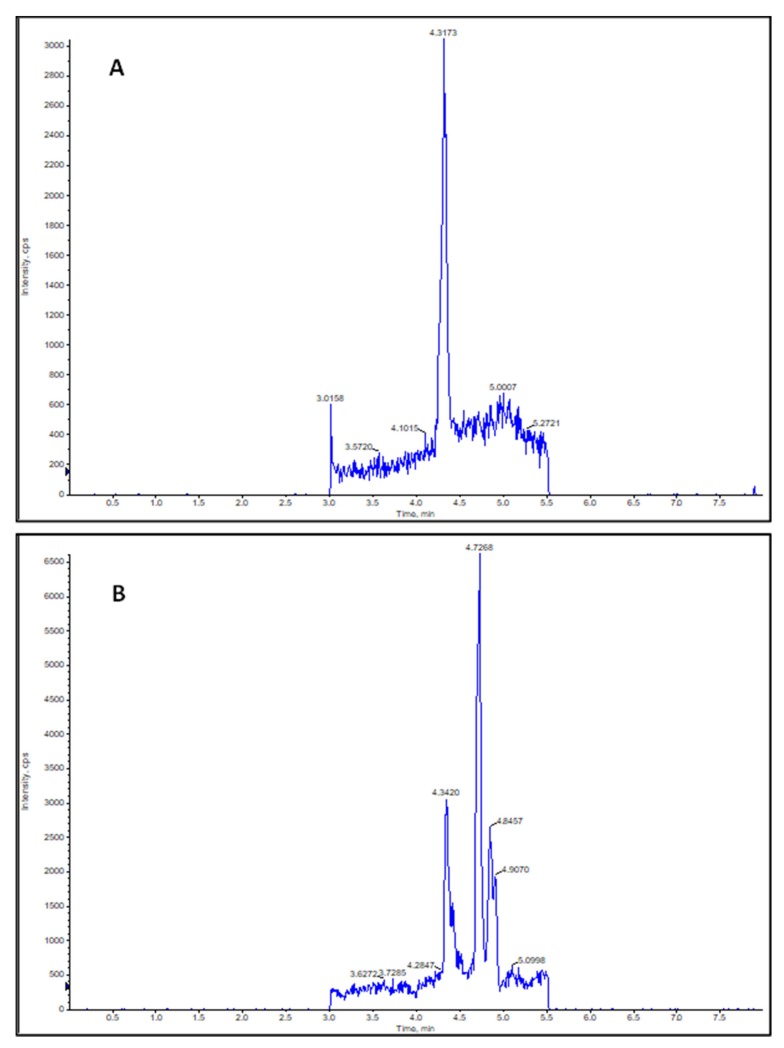
(**A**) Ion Chromatogram of *m*/*z* 326.5 of NO_2_-OA working solution (100 ng/mL). Peak retention time (RT) = 4.3173 min. (**B**) Ion Chromatogram of *m*/*z* 326.5 of a sample derived from 7- day- old *Brassica napus* seedlings. Peak retention time (RT) = 4.3420 min.

Seeds and seedlings of *Brassica napus* are remarkably rich in the unsaturated fatty acid, oleic acid, which implies the possibility of the presence of NO_2_-OA in *Brassica* as well. Figure 3A represents *Brassica napus* seeds and seedlings at day 0, and 2nd, 4th, and 7th day after sowing. Therefore, we performed the analyses of NO_2_-OA concentrations and the quantitative data are presented in Table 1, while the mean NO_2_-OA concentrations with standard errors are shown in Figure 3. Ion chromatograms of the reference standard NO_2_-OA and of the 7-day-old *Brassica napus* seedlings are presented in Figure 4. Further chromatograms are presented as Figure S1. In samples, one further unidentified isomer of NO_2_-OA can be detected with higher retention time.

*Brassica napus* seeds contained a notably high amount of NO_2_-OA compared to seedlings (Table 1, Figure 3B). During the early phase of seedling growth (2nd day), the high NO_2_-OA content of *Brassica* seeds decreased by 78%, and it continued to decline in the following two days (4th day). At the 7th day of seedling growth, the seedlings showed an increased NO_2_-OA content compared to the 2- and 4-day-old seedlings, but it was only approximately 40% of the NO_2_-OA content of the seed. Separated analysis of the shoot and root material indicated that both organs of 7-day-old *Brassica napus* seedlings contained NO_2_-OA in similar quantities (Table 1). In *Arabidopsis* tissues, NO_2_-OA could not be detected, but NO_2_-Ln content was 2.9-fold higher in the seeds than in the 14-day-old seedlings ([6], Table 2). The relatively high NO_2_-Ln and NO_2_-OA contents of *Arabidopsis* and *Brassica* seeds (Table 1, Table 2) indicate the involvement of NO_2_-FA in seed germination possibly as endogenous •NO donors [16]. To examine this hypothesis, we detected •NO levels in *Brassica* seeds and seedlings (2nd, 7th days) (Figure 3C). Compared to seeds, on the 2nd day the •NO content increased by almost 5-fold. The opposite changes in NO_2_-OA and •NO levels suggest that following the induction of germination NO_2_-OA as endogenous donor in the seed may release NO resulting in the decrease in its own free level and concomitant •NO accumulation. Furthermore, it is important to note, that the measured NO_2_-OA concentrations in *Brassica* tissues are in the nanomolar range (Table 1, Figure 3B), that is an order of magnitude higher than the picomolar NO_2_-Ln concentrations of *Arabidopsis* seedlings, cell suspension, pea roots, leaves and rice leaves ([6,7], Table 2). In case of NO_2_-OA supplemented tomato cell suspension, the endogenous NO_2_-OA content was similar to that of the untreated *Brassica napus* seeds and seedlings ([8,9], Table 2). From the above comparisons, it can be concluded that the oleic acid content of *Brassica napus* seeds and seedlings is susceptible to physiological nitration making the plant a rich source of NO_2_-OA.

### 2.4. Exogenous NO_2_-OA Treatment of Brassica Seeds Positively Influences •NO Levels and Germination Capacity

**Figure 5:**
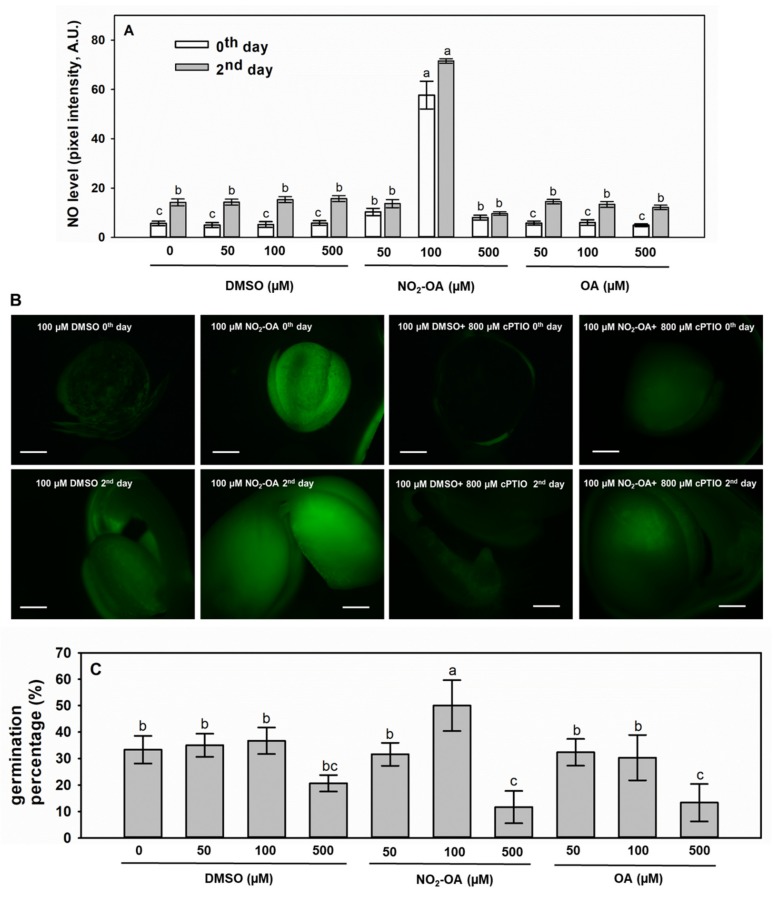
Exogenous NO_2_-OA increases endogenous •NO levels and triggers germination of *Brassica napus* seeds. (**A**) Nitric oxide level in seeds (day 0 of germination) and seedlings (2nd day after sowing) of *Brassica napus* treated for 24 h with 50, 100 or 500 µM NO_2_-OA or with equal concentrations of OA. As controls, seeds were treated with 0, 50, 100 or 500 µM DMSO. Different letters indicate significant differences according to Holm–Sidak test (P ≤ 0.001). (**B**) Representative fluorescent microscopic images of DAF FM-stained *Brassica* seeds and seedlings treated with 100 µM NO_2_-OA or 100 µM DMSO for 24 h in the presence or absence of 800 µM cPTIO. Bars = 800 µm. (**C**) Germination percentage (determined at the 2nd day after sowing) of *Brassica* seeds treated with 50, 100, or 500 µM NO_2_-OA or with equal concentrations of OA. As controls, seeds were treated with 0, 50, 100, or 500 µM DMSO. Different letters indicate significant differences according to the Holm–Sidak test (P ≤ 0.001).

The 24-hour-long treatment with 100 µM NO_2_-OA caused 10-fold increment in the endogenous •NO level of seeds (Figure 5A,B), while NO_2_-OA at 50 µM or 500 µM concentrations did not cause significant changes in •NO levels compared to controls (50 or 500 µM DMSO, respectively). The significant effect of 100 µM NO_2_-OA on •NO level prevailed also on the 2nd day after sowing, since the •NO level of NO_2_-OA-supplemented seedlings was 4.7 times that of the control (100 µM DMSO, Figure 5A,B). The NO_2_-OA-induced DAF-FM fluorescence was quenched by the •NO scavenger cPTIO (Figure 5B) indicating that the alterations in DAF fluorescence correspond to •NO level changes. Oleic acid (OA) treatments applied as controls did not influence •NO levels either in seeds or in 2-day-old seedlings (Figure 5A).

The germination percentages were altered in accordance with •NO levels, since 100 µM NO_2_-OA treatment doubled the percentage of germinated seeds compared to control (100 µM DMSO) (Figure 5C). DMSO alone at 50 µM or 100 µM concentrations had no effect on seed germination, although in the presence of 500 µM DMSO germination percentage of *Brassica* seeds slightly decreased compared to control (0 µM DMSO). Moreover, OA at 50 or 100 µM concentration exerted no effect on germination of *B. napus* seeds (Figure 5C). The effect of DMSO could dominate in case of 500 µM NO_2_-OA or 500 µM OA treatments, since in these seeds the germination ability was weaker than in control and •NO levels remained at control level (Figure 5A). These data clearly indicate that exogenous NO_2_-OA can result in the long-term increase of endogenous •NO level in *Brassica napus* seeds and seedlings, i.e., it may act as a •NO donor. The fact that nitro-fatty acids may act as •NO donors in plants has been previously raised [16]. It was revealed that NO_2_-Ln has the ability to release in vivo, in leaves and roots of old *Arabidopsis* plants [26], in *Arabidopsis* seedlings [16], and in *Arabidopsis* cell-suspension cultures [27]. Contrary to NO_2_-Ln and to NO_2_-OA in our study, exogenous application of NO_2_-OA (0.5, 5, 10, 12.5, 25, or 50 µM, 1 h or 6 h) did not increase •NO level in tomato cell suspension as was recently reported by Di Palma et al. [8,9]. Contradiction of the results can be explained by that here higher concentration of NO_2_-OA (100 µM) were applied for longer time period (24 h) than in the studies of Di Palma et al. [8,9].

### 2.5. NO_2_-OA Releases •NO in Vitro

**Figure 6:**
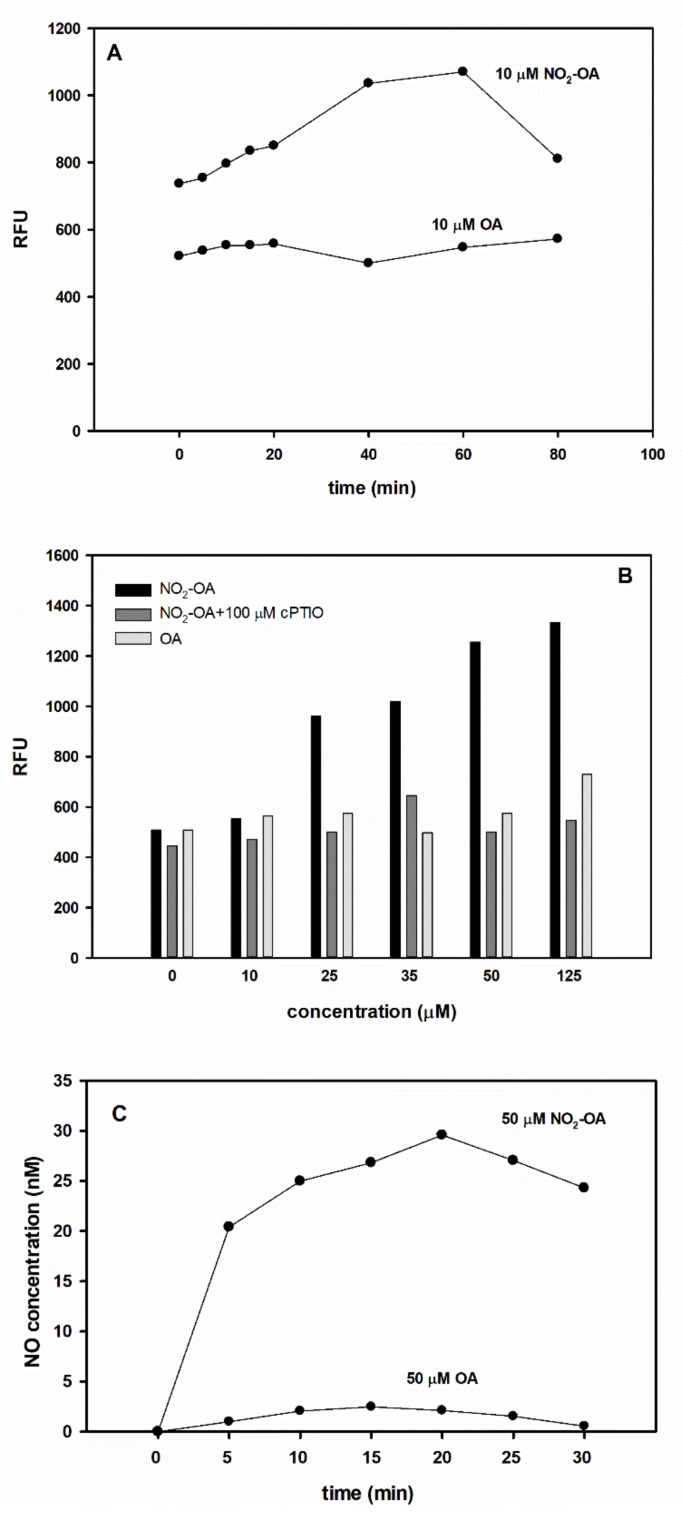
NO_2_-OA liberates •NO in solutions (**A**) Relative fluorescence units (RFU) of DAF-FM fluorescence in solutions in the presence of 10 µM NO_2_-OA or 10 µM OA measured at different time points. (**B**) Relative fluorescence units (RFU) of DAF-FM fluorescence in solutions containing different concentrations of NO_2_-OA (with or without 100 µM cPTIO) or OA. Data were recorded after 20 min incubation. (**C**) •NO concentration (nM) measured by ISO-NOP (2 mm) electrode in solutions containing 50 µM NO_2_-OA or OA measured at different time points.

Additionally, the •NO donor nature of NO_2_-Ln was proved by using several different in vitro approaches (DAF fluorescence, oxyhaemoglobin method, ozone chemiluminescence, [27]. In this work, we performed in vitro tests to support the hypothesis regarding the •NO donor role of NO_2_-OA (Figure 6). Spectrofluorometric measurement of DAF-FM-associated fluorescence revealed that the sample containing 10 µM NO_2_-OA liberated a relatively small amount of •NO during the 80 min period, while the same dosage of OA did not induce •NO level increase compared to the blind sample containing only buffer and the fluorophore (Figure 6A). Moreover, NO_2_-OA liberated •NO in a concentration-dependent manner and the fluorescence increase could be quenched by cPTIO. Elevating doses of OA did not increase •NO levels (Figure 6B).

Using the more sensitive •NO electrode, we could quantify •NO liberation in NO_2_-OA solution (pH 5.8) within 5 min reaching its maximum (~30 nM •NO) after 20 min incubation. The produced •NO concentrations (20–30 nM) quantified by ISO-NOP electrode in this study are similar to those measured by •NO autoanalyzer in the case of 100 µM NO_2_-Ln or 80 µM NO_2_-LA [27]. The same concentration of OA showed no relevant •NO releasing capacity in the solution (Figure 6C). These in vitro data support the •NO donor character of NO_2_-OA in solutions and the degree of •NO liberation is similar to other NO_2_-FAs. However, the same dosage of S-nitrosoglutathione (GSNO) or sodium nitroprusside (SNP) produced approx. 10-fold higher •NO concentration in solutions following 20 min incubation in light (data not shown) indicating that NO_2_-FAs (including NO_2_-OA) •NO donor capacity is much lower compared to “classical •NO donors”.

In our experimental system, NO_2_-OA (100 µM, 24 h) treatment of *Brassica* seeds also promoted germination, presumably through the induction of high •NO levels. In vitro tests revealed that the concentration of liberated •NO is relatively low (Figure 6), but in seeds NO_2_-OA treatment caused intense •NO formation (Figure 5). Therefore, we assume that beyond direct •NO emission, secondary signal processes may also be activated in the presence of NO_2_-OA leading to the activation of •NO metabolic routes in seeds and seedlings. It is well known, that •NO attenuates seed dormancy and promotes germination and we are beginning to recognize also the molecular mechanisms of •NO action [28].

## 3. Materials and Methods

### 3.1. Plant Material and Growing Conditions

Experiments were carried out on *Brassica napus* L. (cv. GK Gabriella) seedlings. The seeds were obtained from the Cereal Research Non-profit Ltd., Szeged, Hungary. *Brassica* seeds were surface sterilised in 70% (v/v) ethanol and 5% (v/v) sodium hypochlorite, then placed on moistened filter paper in Petri dishes (9 cm diameter, 30 seeds/Petri dish). Germination took place under controlled conditions (150 µmol m^−2^ s^−1^ photon flux density, 12 h/12 h light/dark cycle, relative humidity 55–60% and temperature 25 ± 2 °C). For the NO_2_-OA analysis, the samples were taken at the 2nd, 4th and 7th day after sowing. Additionally, seeds after imbibition, at the early stage of germination (day 0) were also sampled for NO_2_-OA quantification. Plant material (5 g) was collected, frozen in liquid nitrogen and stored at –80 °C until the analyses.

### 3.2. Synthesis and Structure Determination of 9-Nitro-Oleic Acid Standard

Using the slightly modified method of Woodcock et al. [29], 9-nitro-oleic acid was synthesized. Bromononanoic acid was used as starting material for allylization to gain 9-bromononanoic acid allyl ester. This compound was nitrated using silver nitrite. In the next step, 10-hydroxy-9-nitro-octadecanoic acid allyl ester was synthesized by the addition of nonyl aldehyde and 1,8-diazabicyclo[5.4.0]undec-7-ene. This compound was acylated with acetic anhydride in the presence of *p*-toluenesulfonic acid to gain 10-acetoxy-9-nitro-octadecanoic acid allyl ester. Deacylation in the presence of Na_2_CO_3_ led to the synthesis of 9-nitro-oleic acid allyl ester. The final product 9-nitro-oleic acid was gained by a catalytic hydrolysis using palladium tetrakis(triphenylphosphine). Purification was carried out by flash chromatography on silica gel (Merck, 40–63 µm) using a gradient of 0.5% acetic acid. Fractions with similar compositions were combined and the combined fraction containing a spot with remarkable absorption at 254 nm and with an R_f_ approximately 0.5 (silica gel plate, eluent CHCl_3_–MeOH 95:5) was subjected to NMR identification and purity check.

^1^H (500.1 MHz) and ^13^C (125.6 MHz) NMR spectra were recorded in CDCl_3_ on a Bruker Avance DRX-500 spectrometer. The peaks of the residual solvent were taken as reference points. The compound was identified by comparison of its chemical shifts with literature data [25,30]. Mass spectrometric identification was performed on an API 2000 triple quadrupole tandem mass spectrometer (MDS Sciex, Toronto, ON, Canada) equipped with electrospray ion source. Mass spectrometric measurement was carried out by direct infusion in Q1 MS scan type in negative mode. Flow rate was set to 40 µL/min, scan range was set from *m*/*z* 100 to 1000, ion source temperature was set to 100 °C. The nebulizer gas was set to 18 psi. Measured molecule ion mass shown to be *m*/*z* 326.5, calculated neutral molecule mass was 327.4589 Da.

### 3.3. LC-MS Quantification of NO_2_-OA in Brassica Seeds and Seedlings

For the quantification of NO_2_-OA, 5 g of fresh plant material (seeds, whole seedlings or separated root and shoot) was used and the analysis was conducted by LC-MS. Mass spectrometry measurement was performed using single ion monitoring (SIM) on an API 2000 triple quadrupole tandem mass spectrometer (MDS Sciex, Toronto, ON, Canada) equipped with electrospray ion source. The nebulizer and heater gas was nitrogen, generated from a Peak NM20Z nitrogen generator (Peak Scientific Instruments Ltd., Scotland, UK) coupled with an Atlas Copco SF 4FF compressor. The nebulizer gas was set to 30 psi, the heater gas was set to 80 psi. The ion source temperature was set to 300 °C. Measurement was in negative mode. The voltage volumes were adjusted to *m*/*z* 326.5, the collision energy was set to −5 V, focusing potential to −330 V, declustering potential to −61 V and entrance potential to −10 V. HPLC separation was performed with Shimadzu HPLC system (Kyoto, Kyoto Prefecture, Japan): DGU-20A3 degasser, CBM-20A controller, two LC-20AD pumps, SIL-20A HT autosampler, CTO-20AC column thermostat, SPD-20A UV-Vis detector, using Kinetex F5 (100 × 4.6 mm, 2.6 µm, 100Ä) (Phenomenex, Inc., Torrance, CA, USA). Elution was carried out with the gradient system of H_2_O–methanol (0 min: H_2_O–methanol 2:8, 0.6 min: 2:8, 1.5 min: 0:1, 3 min: 0:1, 3.2 min: 2:8, 8.2 min: 2:8). The oven was set to 40 °C and the flow rate was 600 µL/min. The retention time of NO_2_-OA was 4.32 min. Data acquisition was performed with Analyst software (ver. 1.6.3). Quantification of nitro-oleic acid was carried out by using the synthesized standard (see in Section 2.2).

Plant material was extracted with pure MeOH (HPLC grade) in a VWR Ultrasonic Cleaner USC 300D (capacity: 1 L, internal dimension: W × D × H 240 × 135 × 100 mm) ultrasonic bath for 10 min, with dual half-wave sound with sweep, frequency was set to 45 kHz, ultrasonic power to 80 W, temperature was set to 25 °C, and filtered through a syringe filter (PTFE, 0.45 µm pore size, Labex Ltd.), the first half ml of the filtrate was thrown into the waste. From each plant part, three parallel samples were prepared, and each sample was injected three times.

### 3.4. •NO Detection in Brassica napus Seeds and Seedlings

Nitric oxide levels were examined in seeds as well as in 2- and 7-day-old seedlings of *Brassica napus* using the 4-amino-5-methylamino- 2′,7′-difluorofluorescein diacetate (DAF-FM DA) according to Kolbert et al. [31]. Plant samples were incubated in 10 µM dye solution for 30 min (darkness, 25 ± 2 °C) and washed twice with Tris-HCl (10 mM, pH 7.4). Microscopic analysis was accomplished under Zeiss Axiovert 200 M inverted microscope (Carl Zeiss, Jena, Germany) equipped with a high-resolution digital camera (AxiocamHR, HQ CCD, Carl Zeiss, Jena, Germany) and filter set 10 (exc.: 450–490, em.: 515–565 nm). Pixel intensities were measured on digital photographs (at least 10 photographs per sample per experiment) using Axiovision Rel. 4.8 software (Carl Zeiss, Jena, Germany).

### 3.5. NO_2_-OA Treatment of Brassica napus Seeds

The synthesized NO_2_-OA or OA was dissolved in dimethyl sulfoxide (DMSO) in order to obtain a stock solution (10 mM). The NO_2_-OA or OA stock was diluted with distilled water to the final concentrations (50, 100, 500 µM). Control solutions were prepared by measuring the volume of DMSO corresponding to the stock solutions (indicated as 0, 50, 100, or 500 DMSO). *Brassica napus* seeds (30 seeds per treatment) were incubated in NO_2_-OA, in OA or in control solutions for 24 h on an orbital shaker and then were placed on moist filter paper in Petri dishes (20 seeds/Petri dish). Sets of seeds were treated with NO_2_-OA or control solutions in the presence of 800 µM 2-(4-carboxyphenyl)-4,4,5,5-tetramethylimidazoline-1-oxyl-3-oxide (cPTIO). Germination took place as indicated above. Nitric oxide levels were detected in seeds prior to germination (day 0), and in seedlings 2 days after sowing (2nd day). Germination percentages (%) were also calculated.

### 3.6. Spectrofluorometric Determination of •NO Levels

•NO liberation from NO_2_-OA solutions were detected by fluorescence spectrophotometry (Ref. [27] with modifications). Reaction mixtures (2 mL final volume) containing 0 (blind) 10, 25, 35, 50, 125 µM NO_2_-OA or the same concentrations of OA, plus 2 µM DAF-FM and Tris-HCl (pH 7.4) buffer were incubated at room temperature in the dark for several time periods (5–80 min) and the emitted fluorescence was recorded by a spectrofluorimeter (Hitachi F-4500, Hitachi Ltd., Tokyo, Japan). Excitation wavelength was set at 485 nm and emissions were measured at 515 nm. The NO_2_-OA-induced fluorescence was quenched by the addition of 100 µM cPTIO.

### 3.7. Measurement of •NO Concentration by •NO-Specific Electrode

The •NO -sensitive electrode (ISO-NOP, 2 mm, World Precision Instruments Inc., Sarasota, FL, USA) was calibrated using a method based on S-nitroso-N-acetylpenicillamine (SNAP) decomposition to •NO in the presence of copper [32]. Two mL of NO_2_-OA or OA (both at 50 µM concentration) solutions were prepared in a 5-mL glass bottle and were measured immediately after preparation. To ensure constant mixing of the solution a magnetic stirrer was applied during the measurement. •NO concentration (nM) was calculated from a standard curve.

### 3.8. Statistical Analysis

All results are shown as mean ± SE. Data were statistically evaluated by the Holm–Sidak method (One-way ANOVA, P ≤ 0.001) using SigmaPlot 12.

## 4. Conclusions

Following successful standard synthesis and method optimization, we have been the first to observe that *Brassica* seeds and seedlings contain free NO_2_-OA. Exogenous treatment of *Brassica* seeds with NO_2_-OA promoted germination and increased endogenous •NO level suggesting that NO_2_-OA may be involved in germination as an •NO donor. The •NO liberating capacity of NO_2_-OA was proved also by in vitro approaches (spectrofluorometric detection of DAF-FM fluorescence and •NO-sensitive electrode). Due to their relatively high NO_2_-OA concentrations, *Brassica* sprouts can be considered as a good source of dietary NO_2_-OA intake in addition to their nutrient, mineral and vitamin content. Therefore, future studies should quantify the NO_2_-OA content of additional *Brassica* species and food plants.

## Figures and Tables

**Table 1:** Concentrations of nitro-oleic acid (NO_2_-OA) in *B. napus* seeds and differently developed seedlings. N/A (not available) indicates data under the detection limit. Samples were injected three times, the precision (RSD, %) of the sample measurements ranged between 0.1348–3.9822 RSD%. The precision between the parallel samples ranged between 9.4828–26.6930 RSD%.

Sample	NO_2_-OA Concentration (nmol/g Fresh Weight)	Mean NO_2_-OA Concentration (nmol/g Fresh Weight)	RSD (%)
Seed 1	1.4582	1.6987	12.2753
Seed 2	1.8293		
Seed 3	1.8086		
Seedling 2nd day 1	0.4378	0.3701	16.1281
Seedling 2nd day 2	0.3475		
Seedling 2nd day 3	0.3250		
Seedling 4th day 1	N/A		
Seedling 4th day 2			
Seedling 4th day 3			
Seedling 7th day 1	0.5569	0.6622	26.6930
Seedling 7th day 2	0.8663		
Seedling 7th day 3	0.5635		
Shoot 7th day 1	0.3124	0.3470	12.2250
Shoot 7th day 2	0.3943		
Shoot 7th day 3	0.3343		
Root 7th day 1	0.4681	0.4260	9.4828
Root 7th day 2	0.3876		
Root 7th day 3	0.4221		

**Table 2:** Concentration values of free NO_2_-FA determined in different plant species and experimental systems (modified from Ref. [7]). Abbreviations: ACSC = *Arabidopsis* cell suspension culture.

Plant Species	Organ or Xperimental System	Type of NO_2_-FA Detected	Concentration of NO_2_-FA Detected (pmol/g Fresh Weight)	Refs.
***Arabidopsis thaliana***	seed	NO_2_-Ln	11.18	[6]
	14-day-old seedling	NO_2_-Ln	3.84	
	30-day-old leaves	NO_2_-Ln	0.36	
	45 day-old leaves	NO_2_-Ln	0.54	
	9-day-old ACSC	NO_2_-Ln	0.28	
***Pisum sativum***	root	NO_2_-Ln	0.072	[7]
	leaf	NO_2_-Ln	0.084	
	mitochondria	NO_2_-Ln	0.282	
	peroxisomes	NO_2_-Ln		
***Oryza sativa***	leaf	NO_2_-Ln	0.748	
***Solanum lycopersicum***	cell suspension treated with NO_2_-OA (0.5, 5, 10, 12.5, 25, 50 µM, 1 h or 6 h)	NO_2_-OA	~2500	[8] [9]

## Supplementary Materials

The following are available online at https://www.mdpi.com/2223-7747/9/3/406/s1. Figure S1: Chromatograms of *B. napus* seeds or seedlings (**A**) Ion chromatogram of *m*/*z* 326.5 of a sample derived from *Brassica napus* seed. (**B**) Ion chromatogram of *m*/*z* 326.5 of a sample derived from 2-day-old *Brassica napus* seedling. (**C**) Ion chromatogram of *m*/*z* 326.5 of a sample derived from 4-day-old *Brassica napus* seedling. (**D**) Ion chromatogram of *m*/*z* 326.5 of a sample derived from 7-day-old *Brassica napus* shoot. (**E**) Ion chromatogram of *m*/*z* 326.5 of a sample derived from 7-day-old *Brassica napus* root.
