# Supplementary material for: Nitro-Oleic Acid in Seeds and Differently Developed Seedlings of Brassica napus L
Source: Plants (Basel). 2020 Mar 24;9(3):406. doi: 10.3390/plants9030406 (PMC7154869; doi:10.3390/plants9030406)
Supplement: Supplementary file 1 [file plants-09-00406-s001.pdf]

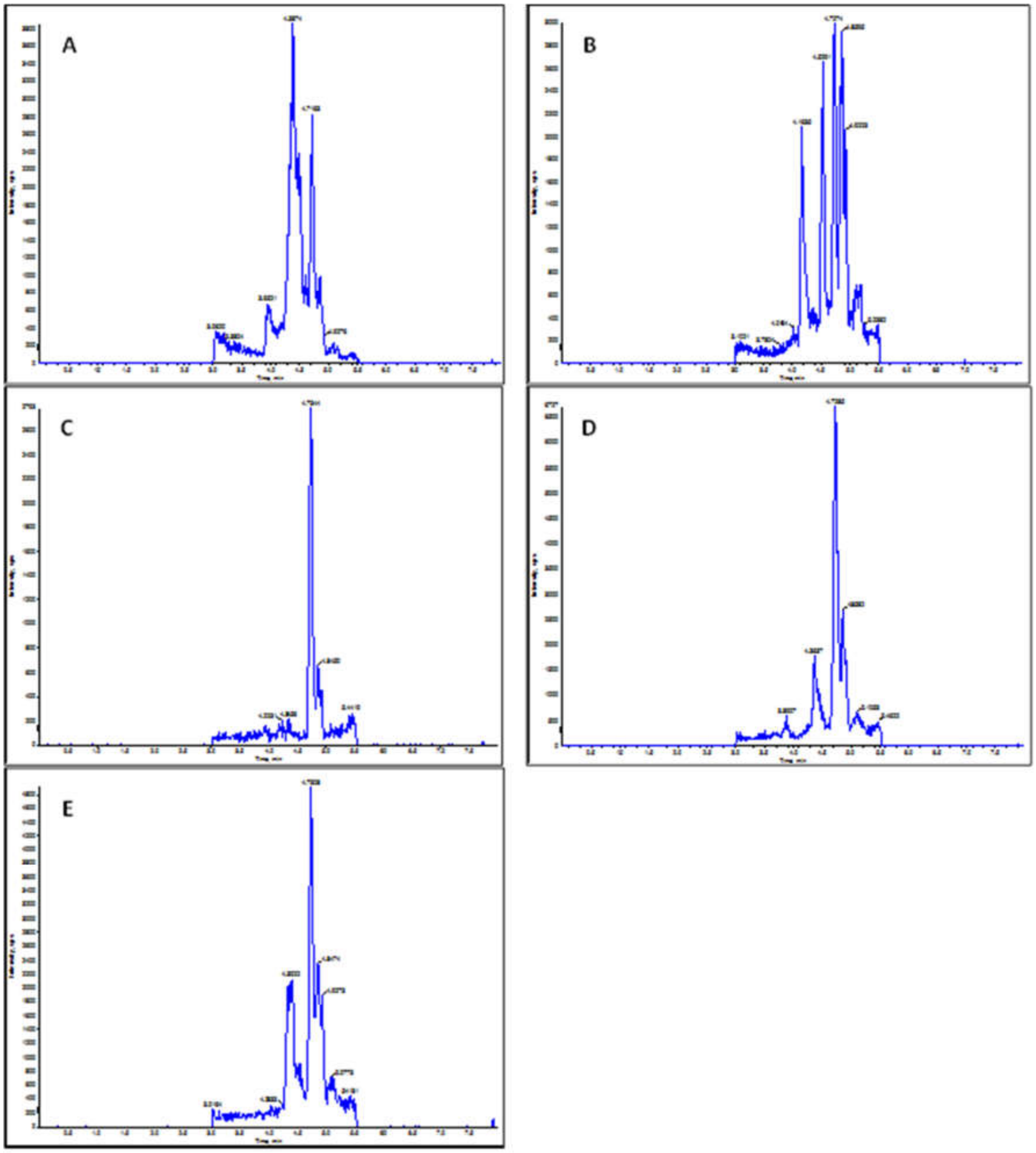

Fig S1 Chromatograms of *B. napus* seedlings (A) Ion chromatogram of m/z 326.5 of a sample derived from *Brassica napus* seed. (B) Ion chromatogram of m/z 326.5 of a sample derived from 2-day-old *Brassica napus* seedling. (C) Ion chromatogram of m/z 326.5 of a sample derived from 4-day-old *Brassica napus* seedling. (D) Ion chromatogram of m/z 326.5 of a sample derived from 7-day-old *Brassica napus* shoot. (E) Ion chromatogram of m/z 326.5 of a sample derived from 7-day-old *Brassica napus* root.
